# Addictive behaviors among prisoners: neuropsychological perspectives on risk, resilience, and intervention response

**DOI:** 10.3389/fpsyg.2026.1770632

**Published:** 2026-03-04

**Authors:** Zalmizy Hussin

**Affiliations:** School of Applied Psychology, Social Work and Policy, College of Arts and Sciences, Universiti Utara Malaysia, Sintok, Kedah Darul Aman, Malaysia

**Keywords:** executive function, intervention response, neuropsychology, prisoners, substance use disorders

## Introduction

Addiction is a major concern among incarcerated individuals and poses challenges for correctional systems, public health, and psychological services. People entering prison have very high rates of substance use disorders, often co-occurring with psychiatric comorbidities, histories of marginalization, and early-life adversity (Favril et al., 2025; Jones et al., 2025; Syasyila et al., 2025). Recent global estimates suggest that the prevalence of substance use disorders (SUDs) among prisoners is substantially higher than in the general population, with some studies indicating that up to half of incarcerated individuals meet diagnostic criteria (Favril et al., 2025). Despite advances in addiction science, responses to substance use in custodial settings remain inconsistent and often prioritize institutional control over factors known to shape vulnerability and recovery.

Modern neuropsychological models propose that SUDs involve dysregulated executive control, reward learning, and stress responsiveness (Lopes et al., 2021; Ribeiro et al., 2025). Developmental research similarly highlights how variation in neurocognitive development and environmental contexts helps explain differences in risk, resilience, and treatment response (Conrod and Nikolaou, 2016). For many individuals, substance use begins in adolescence or young adulthood, when brain systems are particularly sensitive to adversity and reward (Nusslock et al., 2024; Hoffmann and Hoffmann, 2025).

In this opinion piece, we extend these perspectives by hypothesizing that substance addictive behaviors among incarcerated individuals are best conceptualized within a neuropsychological framework that emphasizes risk, resilience, and heterogeneity in intervention response. Translating insights from adolescent substance use research to corrections may provide a mechanistic understanding of persistent substance use and variable treatment outcomes, framing these patterns as expressions of neurodevelopmental and psychosocial functioning rather than as evidence of moral deficits or wilful non-compliance.

## Neuropsychological risk in prisons

Risk factors for SUDs particularly early trauma and disrupted education along with psychiatric symptoms and chronic stress exposure, intersect in incarcerated populations (Bahji, 2024; Nusslock et al., 2024). These factors influence neural systems that support impulse control, emotion regulation, and reward responsiveness across development (Meredith and Silvers, 2024; Muzik and Diwadkar, 2025; Raji et al., 2025).

Executive-function disturbances are especially important. Reduced inhibition, attention, and cognitive flexibility are common among people with substance use problems and are also frequently observed in incarcerated populations (Inozemtseva and Mejía Núñez, 2019). Prison environments demand high levels of behavioral regulation while offering low levels of autonomy; this mismatch between cognitive capacity and institutional demands can be destabilizing. Neuropsychologically, such a discrepancy may increase reliance on habitual or automatic coping responses, including substance use, in the face of distress.

**Figure 1:**
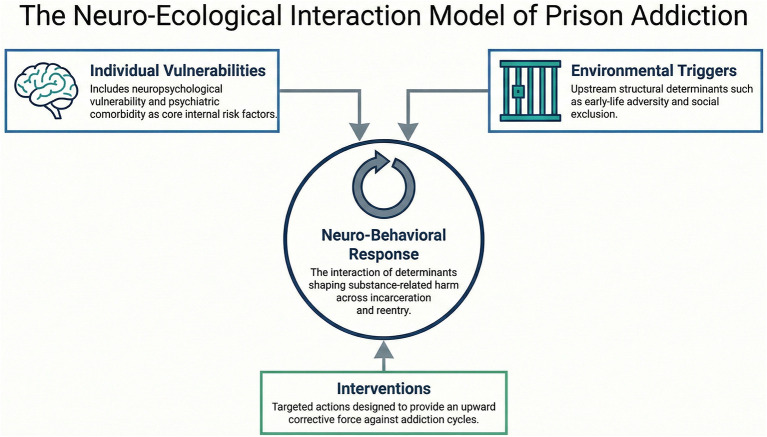
The Neuro-Ecological Interaction Model of Prison Addiction illustrating how individual neuropsychological vulnerabilities and environmental triggers interact with intervention processes to shape neuro-behavioral responses related to substance use across incarceration and community reentry.

Stress mechanisms further amplify risk. Chronic stress engages neurobiological pathways that increase craving and shift decision-making toward immediate relief (Aggarwal et al., 2025; Mehr et al., 2025). Imprisonment may also sustain stress through social threat, uncertainty, and limited coping resources. When substance use functions to reduce negative affect, it may be maintained through negative reinforcement rather than reflecting an intentional rule violation. These interacting neuropsychological and contextual risk processes are summarized in Figure 1, which situates SUDs in incarcerated settings within broader developmental, psychosocial, and structural frameworks.

## Developmental trajectories and continuity of risk

Studies of adolescent substance use highlight adolescence as a developmental period characterized by elevated reward sensitivity alongside immature executive control systems (Castellanos-Ryan et al., 2025; Lozano Wun et al., 2025). When substance use begins during this period, especially in the context of adversity, it can shape learning and coping tendencies that persist into adulthood.

Research suggests that many people in prison began using substances early and later dropped out of school, experienced family dysfunction, or were exposed to violence. These experiences can contribute to developmental pathways marked by self-regulation difficulties and the use of substances to manage emotional or social functioning. Prisons therefore include many individuals whose addictive behaviors reflect accumulated developmental liabilities, not solely decisions made in adulthood.

Applying developmental constructions to adults with criminal records does not equate them with youth. Rather, it recognizes that neuropsychological development is cumulative and that missed opportunities for early intervention can have lasting consequences. Youth-derived resilience constructs such as adaptive emotion regulation and flexible decision-making remain relevant treatment targets in adult correctional contexts.

## Executive functions and intervention responsiveness

Treatment-response heterogeneity is a defining feature of substance use disorders. Neuropsychological differences in executive control help explain why standard treatments vary in effectiveness across individuals. Many prison-based programs (e.g., cognitive behavioral therapy [CBT] and therapeutic communities) target cognitive and behavioral skills that depend on attention, working memory, and future-oriented planning. When people do not participate in or complete these programs, this is often interpreted as a lack of motivation. A neuropsychological perspective, however, highlights a mismatch between the demands of the intervention and an individual's available cognitive resources. This view supports adapting evidence-based approaches rather than dismissing them.

Interventions that reduce cognitive load or directly target executive deficits such as Cognitive Remediation Therapy (CRT) or Mindfulness-Based Relapse Prevention (MBRP) may be better suited for this population. Accessibility can be improved without compromising theoretical soundness by using cognitive scaffolding, simplified prompts and cues, shorter practice blocks with planned breaks, and structured reinforcement.

## Stress, trauma, and emotion regulation

Experiences of severe or chronic stress, including trauma, are known as significant risk factors for addictive behaviors. Early trauma can sensitize stress-response systems, increasing vulnerability to using substances as a coping mechanism, and is further associated with escalation and eventual dependence. It also has been shown to interfere with SUD treatment response (Wemm et al., 2025). These patterns may persist in prison and may be further exacerbated by sustained stressors in the absence of strong emotion-regulation support. Chronic exposure to stress and trauma has been associated with prefrontal cortical atrophy and amygdala hypertrophy, which can undermine neural systems critical for executive functioning and emotion regulation.

Interventions that focus solely on substance-related behavior, without addressing stress regulation, target only one of the primary drivers of continued use. Incorporating trauma-informed principles and emotion-regulation techniques along with sleep-focused interventions is consistent with contemporary neuropsychology and may improve engagement with core addiction treatment services (Lomas, 2024).

## Social reward, learning, and the prison environment

Substance use in prison does not occur in a vacuum; it operates within specific systems of social reinforcement. In custodial settings, social structures are often rigid, and peer acceptance can become a critical currency for survival and identity. Research on adolescent substance use indicates that peer influence and identity formation are powerful drivers of behavior factors that are likely intensified in the close quarters of incarceration (Heidari et al., 2025; Klimukiene et al., 2026).

In this context, substances may serve a dual function: self-medication for distress and a form of social capital used to navigate inmate hierarchies. From a neuropsychological learning perspective, behaviors that reduce perceived social threat or increase belonging can be rapidly reinforced and become habitual. Consequently, effective intervention requires more than prohibition; it also requires the introduction of competing reinforcers. Expanding access to education, meaningful work, and supervised prosocial activities can provide alternative sources of reward and social status that compete with the immediate reinforcement associated with substance use.

## Discussion

Drug addiction among incarcerated populations is better understood as the product of interacting neurocognitive, developmental, and environmental processes than as a series of isolated behavioral choices. Many individuals enter prison with entrenched substance use trajectories shaped by early adversity, disrupted educational and social pathways, and limited access to timely prevention and treatment. These cumulative experiences can produce enduring alterations in neurobiological systems supporting executive control, stress regulation, and reward learning, increasing vulnerability to substance-related harm across the lifespan (Hinckley et al., 2024). Framing addiction within this broader model aligns with contemporary psychological accounts in which risk and resilience are dynamic and context-dependent, not simply a matter of “good” or “bad” decision-making.

Current correctional responses often fail to reflect this evidence base. Rather than reducing vulnerability, custodial environments can amplify it. Executive-function impairments deficits in inhibition, attention, and cognitive flexibility collide with institutional expectations for sustained behavioral regulation under conditions of restricted autonomy (Lee, 2025). Meanwhile, incarceration-related stressors (threat, uncertainty, and social isolation) can bias decision-making toward immediate relief and habitual coping strategies (Aggarwal et al., 2025; Wemm et al., 2025). In practice, policies that rely primarily on deterrence and punishment disregard how stress physiology, learning processes, and constrained choice architecture shape behavior. As illustrated in Figure 1, developmental vulnerability, psychiatric comorbidity, and contextual stressors converge into a multidimensional risk configuration; continued substance use in prison is therefore more plausibly understood as maladaptive coping under dysregulated internal states than as straightforward defiance.

Treatment heterogeneity further exposes the limits of “one-size-fits-all” programming. Many prison-based interventions assume cognitive and affective capacities that may be compromised in individuals with executive dysfunction and heightened stress reactivity (Ribeiro et al., 2025). Yet when programs yield modest or inconsistent outcomes, non-response is often attributed to poor motivation or limited insight an attribution that is not only weakly supported but also administratively convenient. These framing risks re-labeling neuropsychological constraints as character flaws, thereby legitimizing exclusionary practices (e.g., removal for “non-compliance”) that predictably disadvantage those with the greatest clinical need. A more defensible interpretation is that poor engagement frequently signals intervention misalignment: the cognitive demands, delivery format, and reinforcement structures of standard programs may not match participants' available resources. A structured overview of the mechanisms discussed and their relevance to intervention alignment is summarized in Supplementary Table S1.

The transition from prison to the community further highlights the short-sightedness of policies that treat incarceration as a “reset.” Abrupt changes in structure, social demands, and substance availability interact with stress- and reward-related vulnerabilities, increasing relapse risk and related harms (Syasyila et al., 2025). Approaches that emphasize in-custody compliance without robust continuity of care effectively shift risk into the community, where consequences are often more severe and supports more limited. From a developmental perspective, release should be treated as a predictable continuation of risk, requiring sustained support rather than administrative discharge.

Overall, this analysis argues for moving away from compliance-oriented, deterrence-heavy approaches toward strategies grounded in established psychological science. Integrating neuropsychological mechanisms with developmental and structural perspectives clarifies why prevailing correctional responses are often underpowered: they target visible behavior while neglecting the learning contingencies, cognitive constraints, and stress-related drivers that maintain it. This critique does not deny the importance of safety or accountability; it argues that current policies too often pursue these goals through methods that are clinically mis-specified and ethically costly. A scientifically grounded correctional approach would prioritize intervention–person fit, reduce avoidable stressors, and build continuity of care as core not optional elements of addiction response in prison populations.
