# Supplementary material for: Addictive behaviors among prisoners: neuropsychological perspectives on risk, resilience, and intervention response
Source: Front Psychol. 2026 Mar 4;17:1770632. doi: 10.3389/fpsyg.2026.1770632 (PMC12997179; doi:10.3389/fpsyg.2026.1770632)
Supplement: Supplementary file 1 [file Table_1.docx]

*Addictive Behaviors Among Prisoners: Neuropsychological Perspectives on Risk, Resilience, and Intervention Response*

Supplementary Table S1. Conceptual framework supporting interpretation of addictive behaviors among prisoners

| **Domain** | **Conceptual focus** | **Description** | **Relevance to the main article** |
| --- | --- | --- | --- |
| Executive control | Cognitive regulation | Includes inhibition, attention, working memory, and cognitive flexibility involved in self-regulation and decision-making | Used to interpret variability in treatment engagement and relapse risk without assuming motivational failure |
| Stress regulation | Neuropsychological stress response | Refers to physiological and psychological mechanisms activated by chronic or acute stress, including trauma-related processes | Supports interpretation of substance use as negative reinforcement under custodial stress |
| Reward learning | Reinforcement sensitivity | Encompasses processes governing habit formation, salience attribution, and preference for immediate rewards | Explains persistence of substance use and uneven response to abstinence-based interventions |
| Developmental vulnerability | Lifespan trajectory | Describes how early substance use initiation and adversity shape long-term neuropsychological risk | Justifies translation of youth substance-use frameworks to adult prison populations |
| Resilience mechanisms | Adaptive regulation | Includes emotion regulation, cognitive flexibility, and alternative reinforcement pathways | Frames resilience as a modifiable process rather than a fixed trait |
| Intervention response | Differential responsiveness | Variation in outcomes based on alignment between intervention demands and neuropsychological capacity | Informs interpretation of strengths and limitations of existing correctional treatment models |
| Transition sensitivity | Contextual change | Neurobehavioral impact of abrupt environmental shifts during prison-to-community release | Supports discussion of continuity of care as part of intervention trajectory |

# References

Aggarwal, D., Naik, J., and Lindquist, D. H. (2025). Biphasic model of addiction: Neurobehavioral adaptations. *Curr Behav Neurosci Rep* 12, 25. doi: 10.1007/s40473-025-00314-0

Bahji, A. (2024). Navigating the complex intersection of substance use and psychiatric disorders: A comprehensive review. *JCM* 13, 999. doi: 10.3390/jcm13040999

Castellanos-Ryan, N., Pocuca, N., and Peeters, M. (2025). “Adolescent development and vulnerability for addiction,” in *The Sage Handbook of Addiction Psychology*, eds. I. Franken, R. Wiers, and K. Witkiewitz (1 Oliver’s Yard, 55 City Road  London  EC1Y 1SP: Sage Publications Ltd), 210–225. doi: 10.4135/9781529673913.n14

Favril, L., Strang, J., and Fazel, S. (2025). Drug use among people in prison: A global review of epidemiology, harms and interventions. *Addiction*, add.70245. doi: 10.1111/add.70245

Heidari, M., Zareipour, M., Mataji-Amirroud, M., and Mataji-Amirroud, M. (2025). Exploring peer influence: A phenomenological analysis of adolescent propensities for association with deviant peers and substance use. *Deviant Behavior*, 1–23. doi: 10.1080/01639625.2025.2516637

Hinckley, J., Adams, Z., Dellucci, T., and Berkowitz, S. (2024). Co-occurring trauma- and stressor-related and substance-related disorders in youth: A narrative review. *MRAJ* 12. doi: 10.18103/mra.v12i8.5688

Hoffmann, J. P., and Hoffmann, C. S. (2025). Childhood trauma and adolescent substance use: an integrative perspective. *Future Science OA* 11, 2557763. doi: 10.1080/20565623.2025.2557763

Inozemtseva, O., and Mejía Núñez, E. (2019). “Executive dysfunction associated with substance abuse,” in *Dysexecutive Syndromes*, eds. A. Ardila, S. Fatima, and M. Rosselli (Cham: Springer International Publishing), 123–142. doi: 10.1007/978-3-030-25077-5_6

J. Conrod, P., and Nikolaou, K. (2016). Annual research review: On the developmental neuropsychology of substance use disorders. *Child Psychology Psychiatry* 57, 371–394. doi: 10.1111/jcpp.12516

Jones, A. A., Brant, K., Bishop, R. E., Strong-Jones, S., and Kreager, D. A. (2025). Just an unfair score: Perceptions of gender inequity in the treatment of substance use disorders among women involved in the criminal legal system. *Journal of Substance Use and Addiction Treatment* 169, 209587. doi: 10.1016/j.josat.2024.209587

Klimukiene, V., Laurinavicius, A., Bagdonaite, S., and Sakalauskas, G. (2026). The role of identity formation in explaining dynamic risk factors among incarcerated emerging adults. *Int J Offender Ther Comp Criminol* 70, 71–87. doi: 10.1177/0306624X251329030

Lee, S. (2025). Executive function variability in autism spectrum disorder: subdomains, developmental trajectories, and clinical implications. *International Journal of Developmental Disabilities*, 1–14. doi: 10.1080/20473869.2025.2581644

Lomas, C. (2024). Neurobiology, psychotherapeutic interventions, and emerging therapies in addiction: a systematic review. *Journal of Addictive Diseases*, 1–19. doi: 10.1080/10550887.2024.2440184

Lopes, F. M., Boos, F. Z., Zanini, A. M., and Czermainski, F. R. (2021). “Neuropsychological and behavioral aspects of drug use and abuse: Theory, research, and intervention,” in *Drugs and Human Behavior*, eds. D. De Micheli, A. L. M. Andrade, R. A. Reichert, E. A. D. Silva, B. D. O. Pinheiro, and F. M. Lopes (Cham: Springer International Publishing), 151–170. doi: 10.1007/978-3-030-62855-0_11

Lozano Wun, V., Klein, S. D., Collins, P. F., and Luciana, M. (2025). Within-person imbalance of reward sensitivity and executive functioning across adolescent development: A longitudinal examination of the dual systems model from childhood to adulthood. *Developmental Psychology* 61, 2375–2395. doi: 10.1037/dev0001969

Mehr, S. R., Nakhaei, B., Soleimani, H., Madadlou, S. K., Abbasi Maleky, A., and Abbasi-Maleki, S. (2025). “Addiction and stress: Exploring the reward pathways in brain affected by different drugs,” in *Progress in Brain Research*, (Elsevier), 381–404. doi: 10.1016/bs.pbr.2025.01.012

Meredith, W. J., and Silvers, J. A. (2024). Experience-dependent neurodevelopment of self-regulation in adolescence. *Developmental Cognitive Neuroscience* 66, 101356. doi: 10.1016/j.dcn.2024.101356

Muzik, O., and Diwadkar, V. A. (2025). Human regulatory systems in the age of abundance: A predictive processing perspective. *Annals of the New York Academy of Sciences* 1545, 16–27. doi: 10.1111/nyas.15302

Nusslock, R., Alloy, L. B., Brody, G. H., and Miller, G. E. (2024). Annual research review: Neuroimmune network model of depression: a developmental perspective. *Child Psychology Psychiatry* 65, 538–567. doi: 10.1111/jcpp.13961

Raji, H., Dinesh, S., and Sharma, S. (2025). Inside the impulsive brain: a narrative review on the role of neurobiological, hormonal and genetic factors influencing impulsivity in psychiatric disorders. *Egypt J Neurol Psychiatry Neurosurg* 61, 4. doi: 10.1186/s41983-024-00930-9

Ribeiro, N. S., De Oliveira, I. P., De Almeida, L. F., Silva, P. A. G., and Sartes, L. M. A. (2025). “Interfaces between clinical neuropsychology and cognitive and behavioral therapies in the treatment of drug addiction,” in *Neuropsychology and Substance Use Disorders*, eds. R. A. Reichert, A. L. M. Andrade, and D. De Micheli (Cham: Springer Nature Switzerland), 593–609. doi: 10.1007/978-3-031-82614-6_29

Syasyila, K., Kamaluddin, M. R., Abdullah @ Mohd Nor, H., and Jamir Singh, P. S. (2025). Psychosocial determinants of recidivism risk among incarcerated individuals with a history of substance use: A systematic review. *PLoS One* 20, e0327810. doi: 10.1371/journal.pone.0327810

Wemm, S., Pearlman, M., and Sinha, R. (2025). “Stress and addictive disorders: Drug- and stressor-related effects on stress biological and psychological response,” in *The Sage Handbook of Addiction Psychology*, eds. I. Franken, R. Wiers, and K. Witkiewitz (1 Oliver’s Yard, 55 City Road  London  EC1Y 1SP: Sage Publications Ltd), 145–162. doi: 10.4135/9781529673913.n10
